# Dietary Supplementation with Lactobacilli Improves Emergency Granulopoiesis in Protein-Malnourished Mice and Enhances Respiratory Innate Immune Response

**DOI:** 10.1371/journal.pone.0090227

**Published:** 2014-04-01

**Authors:** Matias Herrera, Susana Salva, Julio Villena, Natalia Barbieri, Gabriela Marranzino, Susana Alvarez

**Affiliations:** 1 Immunobiotics Research Group, Reference Centre for Lactobacilli (CERELA-CONICET), San Miguel de Tucuman, Tucuman, Argentina; 2 Laboratory of Immunobiotechnology, Reference Centre for Lactobacilli (CERELA-CONICET), San Miguel de Tucuman, Tucuman, Argentina; 3 Institute of Applied Biochemistry, Tucuman University, San Miguel de Tucuman, Tucuman, Argentina; 4 INSIBIO-CONICET, National University of Tucuman, San Miguel de Tucuman, Tucuman, Argentina; Institut Pasteur, France

## Abstract

This work studied the effect of protein malnutrition on the hemato-immune response to the respiratory challenge with *Streptococcus pneumoniae* and evaluated whether the dietary recovery with a probiotic strain has a beneficial effect in that response. Three important conclusions can be inferred from the results presented in this work: a) protein-malnutrition significantly impairs the emergency myelopoiesis induced by the generation of the innate immune response against pneumococcal infection; b) repletion of malnourished mice with treatments including nasally or orally administered *Lactobacillus rhamnosus* CRL1505 are able to significantly accelerate the recovery of granulopoiesis and improve innate immunity and; c) the immunological mechanisms involved in the protective effect of immunobiotics vary according to the route of administration. The study demonstrated that dietary recovery of malnourished mice with oral or nasal administration of *L. rhamnosus* CRL1505 improves emergency granulopoiesis and that CXCR4/CXCR12 signaling would be involved in this effect. Then, the results summarized here are a starting point for future research and open up broad prospects for future applications of probiotics in the recovery of immunocompromised malnourished hosts.

## Introduction

In children under 5 years of age, malnutrition is responsible for 54% of the 10.8 million deaths per year and contributes to over half of death (53%) associated with infectious diseases in developing countries [1]. For the surviving children, malnutrition has lifelong implications because it severely reduces a child's ability to learn and grow to their full potential. Thus, malnutrition leads to less productive adults and weaker national economic performance [1]. Adequate and prompt correction of nutritional status is important to reduce morbidity and mortality of infectious diseases associated with malnutrition. However, a simple intervention, such as adequate intake of nutrients, in the presence of recurrent infections, is not enough to reverse this cycle, because infections themselves cause critical loss of protein, energy, vitamins and minerals deposits from the body [2].

In the last years, several investigations aimed to find alternative treatments to accelerate the recovery of immunity and enhance resistance to infections in malnourished hosts. In this regard, developments in the nutrition area suggest that probiotic lactic acid bacteria (LAB) do have benefits in the health of malnourished hosts. Probiotic fermented products play a significant role in the absorption and bioavailability of nutrients, making them particularly beneficial in the feeding of malnourished individuals. In addition, it was demonstrated that probiotics are able to enhance immune function. Thereby, LAB strains with capacity to beneficially modulate the immune system (immunobiotics) represent an attractive safe way to modulate and enhance immune function in immunocompromised malnourished hosts [3], [4].

Previously, we evaluated the effect of several LAB strains on the prevention of *Streptococcus pneumoniae* infection in protein-malnourished mice. Our results showed that the incorporation of some few LAB strains (*Lactobacillus casei* CRL431 or *Lactobacillus rhamnosus* CRL1505) to the repletion diet had a beneficial effect since these treatments accelerated the recovery of the systemic and mucosal immune systems and improved resistance against pneumococcal respiratory infection [5]–[8]. *L. rhamnosus* CRL1505 demonstrated the ability to increase the resistance against pneumococcal infection of immunocompetent and immunocompromised mice [9]–[11]. Moreover, we showed that consumption of a fermented dairy product containing *L. rhamnosus* CRL1505 was associated with a significant decrease of the length and severity of mucosal infections in young children [12].

It is well established that the hematopoietic system in the bone marrow is engaged to generate an appropriate hemato-immune response during infection and inflammation. This process involves direct and indirect instruction of hematopoietic cells by cytokines, chemokines, and conserved patterns of infection, leading to adapted hematopoietic generation and directed migration of cells in need [13], [14]. We performed studies to analyze the extent of the damage induced by malnutrition on B cell development in the spleen and bone marrow and its impact in B cell-mediated immunity in the respiratory tract [11], [15]; and we observed that the number of B220^+^ cells (the whole B cell compartment) was reduced in the bone marrow of malnourished mice [15]. Moreover, protein deprivation induced a significant reduction of pro-B/pre-B (B220^interm^IgM^neg^) and immature B cell (B220^interm^IgM^+^) suggesting that nutritional deprivation leads to the alteration of B-cell development [15]. More recently, we demonstrated that these alterations in B cell development significantly impair the generation of the systemic and respiratory B cell-mediated immunity, conducting to a reduced capacity of malnourished mice to mount an appropriate humoral immune response [11]. We also showed that the addition of *L. rhamnosus* CRL1505 to repletion treatments was able to induce a recovery of B cells and to normalize the numbers of bone marrow immature B220 cells [15], splenic immature B cells and lung mature B lymphocytes [11]. In addition, malnourished mice treated with *L. rhamnosus* CRL1505 were able to establish an improved humoral immune response against the respiratory pneumococcal infection [11].

Previous work indicates that probiotic supplementation is able to improve myelopoiesis in malnourished hosts [15]. However, the capacity of immunobiotic strains to influence myeloid cells development in malnourished hosts and the impact of such effect on innate immunity have not been evaluated in depth. Therefore, in the current study we aimed to: a) evaluate the effect of malnutrition on myeloid cells development in bone marrow and its systemic effect when emergency myelopoiesis is required during the generation of the innate immune response against a respiratory pathogen; b) study the effect of *L. rhamnosus* CRL1505 on emergency myelopoiesis and respiratory innate immune response against *S. pneumoniae* in protein-malnourished mice, in order to gain insight in the knowledge of the mechanism involved in the immunomodulatory effect of the CRL1505 strain. Moreover, considering that nasally administered antigens can induce respiratory and systemic immune responses superior to those obtained using oral stimulation [4], we also aimed to c) comparatively study the effect of orally and nasally administered *L. rhamnosus* CRL1505 in order to establish which is the most effective treatment to improve protection against pneumococcal infection.

## Results

### Protein-malnutrition significantly impairs respiratory and systemic innate immune responses against pneumococcal infection

Malnourished mice were obtained after 21 days of feeding a protein free-diet. Then, mice were replete for 7 days with a balanced conventional diet (BD) or BD supplemented with orally or nasally administered *L. rhamnosus* CRL1505 (BD+LrO and BD+LrN respectively). Malnourished (MNC) and well-nourished (WNC) mice were used as controls.

**Figure 1:**
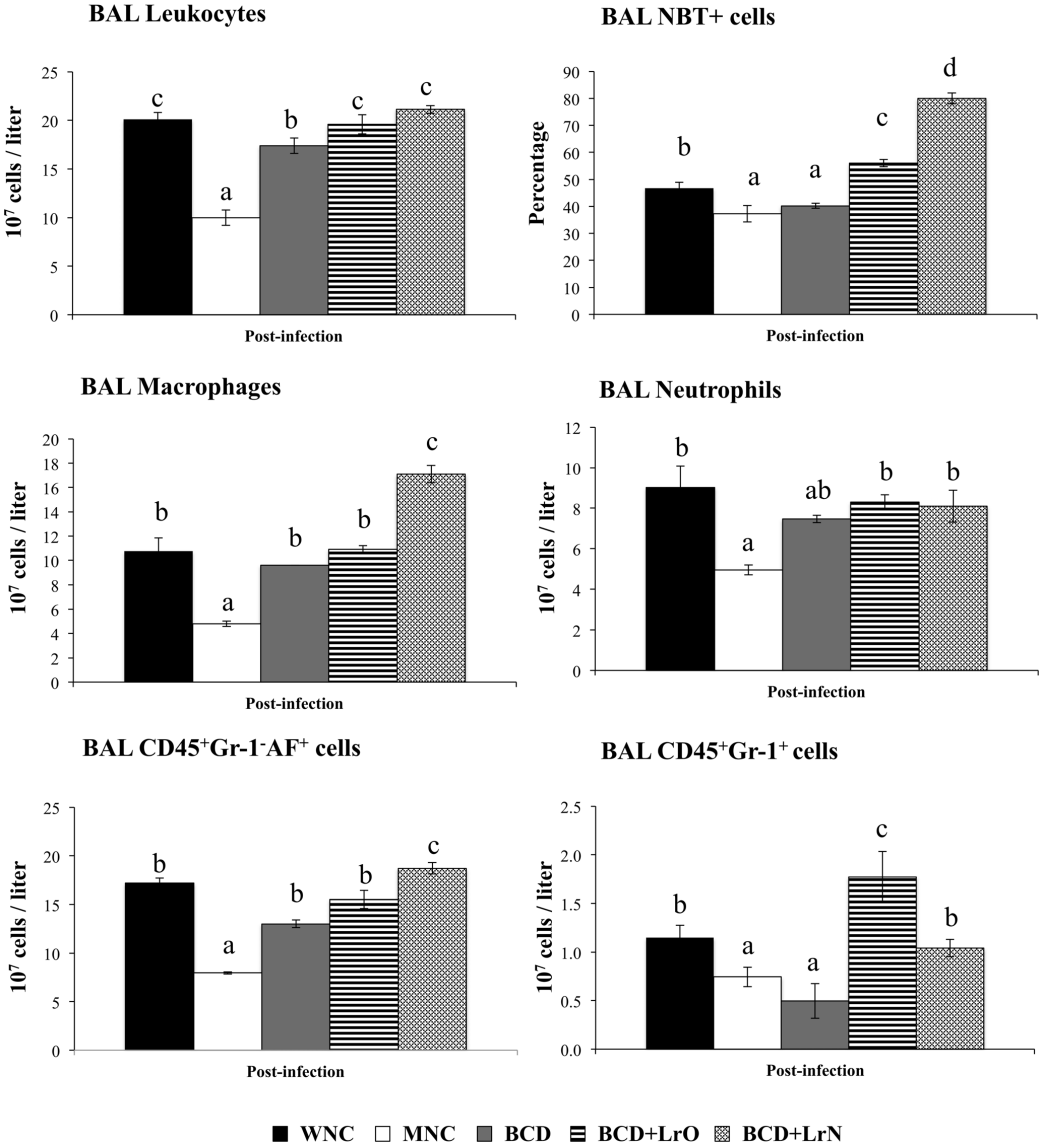
Innate immune cell populations in broncho-alveolar lavages (BAL). Malnourished mice were replete for 7 days with a balanced conventional diet (BD) or BD supplemented with orally or nasally administered *Lactobacillus rhamnosus* CRL1505 (BD+LrO and BD+LrN respectively) and then challenged with 10^5^ cells of *Streptococcus pneumoniae*. Malnourished (MNC) and well-nourished (WNC) mice were used as controls. Leukocytes, macrophages, neutrophils, CD45^+^Gr1^-^AF^+^ cells and CD45^+^Gr1^+^ cells numbers and the percentage of nitroblue tetrazolium (NBT) positive cells were determined after pneumococcal infection (day 2). Values are means for six mice per group with standard deviations represented by verticals bars. Different letters indicate significant differences at the same time point (*p*<0.05) considering a<b<c.

We first studied the number and activity of respiratory phagocytes in both WNC and MNC mice. No differences were observed in number of bronchoalveolar lavages (BAL) leukocytes, neutrophils and macrophages when the two groups were compared (Figure S1). However, as we have previously reported [5], MNC mice showed significantly lower levels of BAL NBT^+^ cells than WNC mice. *S. pneumoniae* infection induced the recruitment of neutrophils and macrophages into the alveoli, resulting in an increase in BAL leukocyte counts on day 2 post-infection (Figure 1). MNC mice showed an impaired recruitment of neutrophils and macrophages since significantly lower numbers of leukocytes, CD45^+^Gr1^-^AF^+^ macrophages and CD45^+^Gr1^+^ neutrophils were found in BAL when compared with WNC mice (Figure 1). In addition to the quantitative alterations of BAL phagocytes, we also observed that malnutrition significantly impaired phagocytic activity of BAL cells (Figure 1).

**Figure 2:**
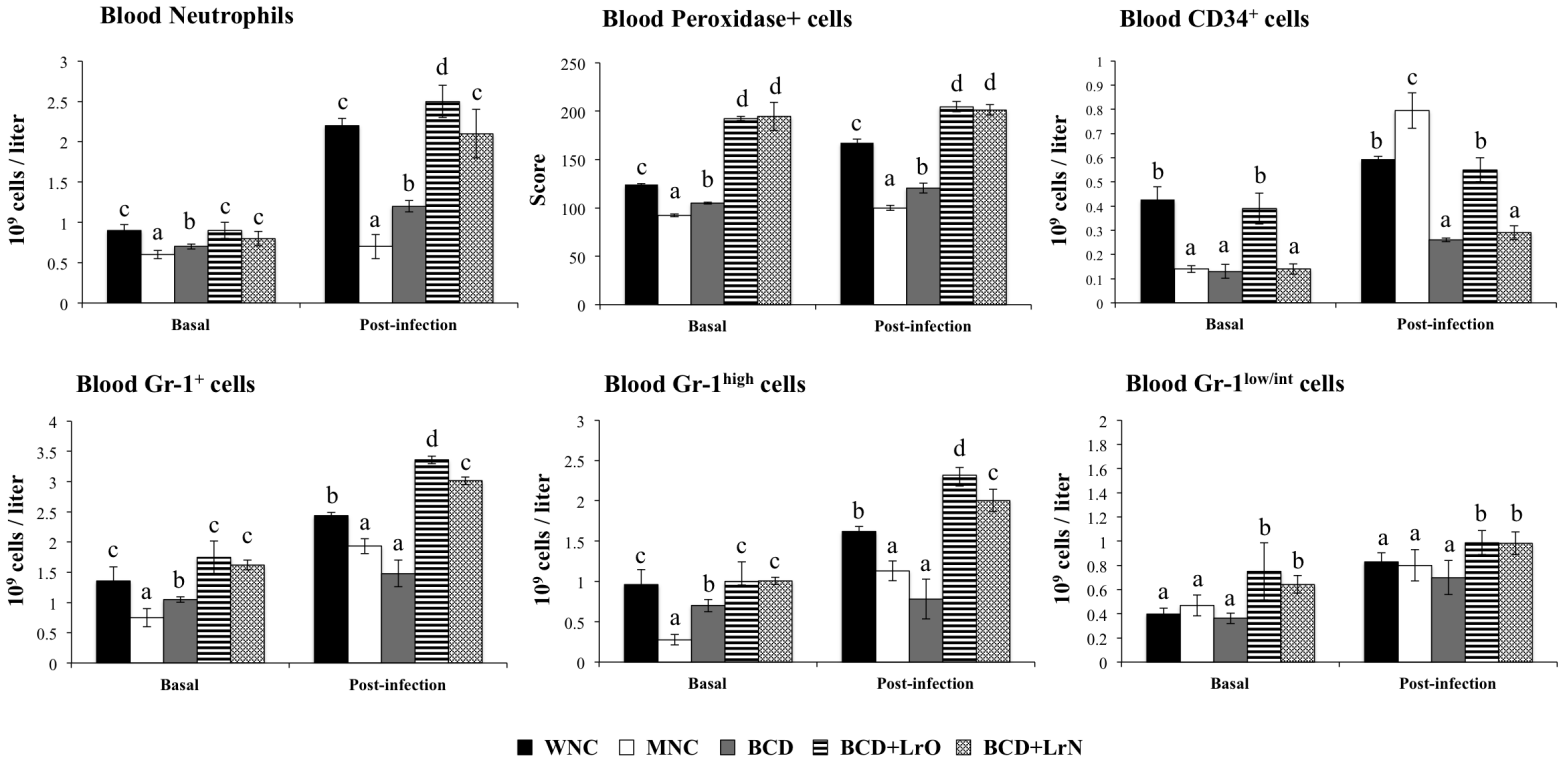
Innate immune cell populations in blood. Malnourished mice were replete for 7 days with a balanced conventional diet (BD) or BD supplemented with orally or nasally administered *Lactobacillus rhamnosus* CRL1505 (BD+LrO and BD+LrN respectively) and then challenged with 10^5^ cells of *Streptococcus pneumoniae*. Malnourished (MNC) and well-nourished (WNC) mice were used as controls. Blood neutrophils, CD34^+^ cells, Gr1^+^ cells, Gr1^high^ cells and Gr1^low/int^ cells numbers and the score of blood neutrophils myeloperoxidase were determined before (basal) and after pneumococcal infection (day 2). Values are means for six mice per group with standard deviations represented by verticals bars. Different letters indicate significant differences at the same time point (*p*<0.05) considering a<b<c.

A decreased number of leukocytes and neutrophils as well as reduced peroxidase scores were detected in MNC mice than in WNC group (Figure S2; Figure 2). We confirmed the impairment of blood neutrophils induced by malnutrition by evaluating the numbers of blood Gr1^+^, Gr1^high^ and Gr1^low^ cells. Significantly lower levels of Gr1^+^ and Gr1^high^ cells were detected in MNC mice while the numbers of blood Gr1^low^ cells were not different from WNC mice (Figure 2). *S. pneumoniae* infection increased blood leukocytes and neutrophils counts, peroxidase scores and blood Gr1^+^ and Gr1^high^ numbers in both experimental groups; however, all these parameters were significantly lower in MNC than in the WNC group (Figure 2). Gr1^low^ cells were also increased by the pneumococcal infection; however, the levels of these cells were equal in both groups (Figure 2). In addition, CD34^+^ blood cells were significantly decreased in MNC (Figure 2). After the challenge with pneumococci, blood CD34^+^ cells were significantly increased in both experimental groups in relation to basal levels. However, MNC mice showed significantly higher numbers of blood CD34^+^ cells than WMC mice (Figure 2).

**Figure 3:**
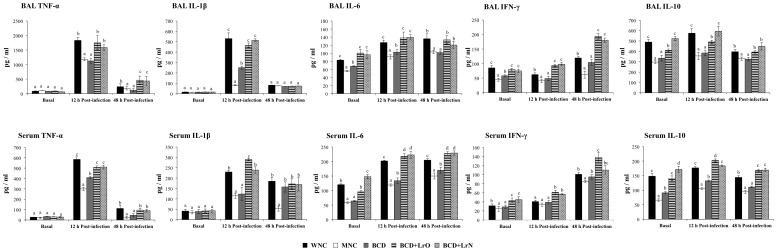
Cytokines in broncho-alveolar lavages (BAL) and blood. Malnourished mice were replete for 7 days with a balanced conventional diet (BD) or BD supplemented with orally or nasally administered *Lactobacillus rhamnosus* CRL1505 (BD+LrO and BD+LrN respectively) and then challenged with 10^5^ cells of *Streptococcus pneumoniae*. Malnourished (MNC) and well-nourished (WNC) mice were used as controls. Levels of BAL and serum TNF-α, IL-1β, IL-6, IFN-γ and IL-10 were determined before (basal) and after pneumococcal infection (hours 12 and 48). Values are means for six mice per group with standard deviations represented by verticals bars. Different letters indicate significant differences at the same time point (*p*<0.05) considering a<b<c.

The levels of TNF-α, IL-1β, IL-6, IFN-γ and IL-10 were studied in serum and BAL samples before the challenge with *S. pneumoniae.* No significant difference on BAL and serum TNF-α and IL-1β was found between WNC and MNC mice (Figure 3). On the contrary, levels of BAL and serum IL-6, IFN-γ and IL-10 were significantly reduced in MNC mice when compered with the WNC group. Challenge with the respiratory pathogen induced an early increase (12 hours post-infection) of TNF-α, IL-1β and IL-6 levels and a late increase of IFN-γ (48 hours post-infection) in BAL and serum, in both experimental groups. However MNC mice showed significantly lower levels of serum and BAL TNF-α, IL-1β, IL-6 and IFN-γ than WNC mice (Figure 3). Infection also increased the levels of the immunoregulatory cytokine IL-10 in serum and BAL of WNC mice; however, MNC mice were not able to increase the production of this cytokine in response to pathogen (Figure 3).

### *L. rhamnosus* CRL1505 administration enhances respiratory and systemic innate immune responses in malnourished mice

We next evaluated the effect of different repletion treatments on blood and BAL cells affected by malnutrition. No significant differences were observed in the number of BAL leukocytes, macrophages and neutrophils between BCD, BCD+LrO and BCD+LrN mice and controls (MNC and WNC mice) (Figure S1). In addition, BCD, BCD+LrO and BCD+LrN mice showed normal values of BAL NBT^+^ cells (Figure S1). After the challenge with *S. pneumoniae*, treatment of malnourished mice with BCD increased the number of BAL leukocytes and CD45^+^Gr1^-^AF^+^ macrophages; however, the treatment was not able to normalize these parameters. Moreover, numbers of BAL CD45^+^Gr1^+^ neutrophils and percentages of NBT^+^ from BCD mice were not different from the MNC group (Figure 1). Treatment with BCD+LrO significantly increased the number of BAL neutrophils and macrophages, as well as phagocytes' activity in response to the infection. In fact BCD+LrO mice showed values of BAL leukocytes and CD45^+^Gr1^-^AF^+^ macrophages that were similar to the WNC group (Figure 1). Furthermore, BAL CD45^+^Gr1^+^ neutrophils and NBT^+^ cells in BCD+LrO mice were significantly higher than WNC mice. In addition, BCD+LrN treatment significantly improved numbers of BAL neutrophils and macrophages and the percentages of BAL NBT^+^ cells. BAL CD45^+^Gr1^+^ neutrophils in BCD+LrN mice showed values that were similar to the WNC group (Figure 1). However, values of BAL CD45^+^Gr1^-^AF^+^ macrophages and NBT^+^ cells in BCD+LrN mice were significantly higher than those observed in the WNC group (Figure 1).

Treatment with BCD slightly increased blood neutrophils numbers, peroxidase scores and Gr1^+^ and Gr1^high^ cells; however, the values did not reach the levels of WNC mice (Figure 2). On the contrary, repletion with both BCD+LrO and BCD+LrN significantly increased blood leukocytes (Figure S2), neutrophils, Gr1^+^ and Gr1^high^ cells numbers reaching similar values than those observed in WNC mice (Figure 2). Additionally, blood peroxidase activity and Gr1^low^ cells in BCD+LrO and BCD+LrN mice were significantly higher than in WNC mice (Figure 2). After *S. pneumoniae* challenge, malnourished mice treated with BCD showed significantly higher levels of neutrophils and Gr1^high^ cells than MNC mice; however blood peroxidase activity and Gr1^low^ cells were not different from malnourished controls (Figure 2). Treatment with both BCD+LrO and BCD+LrN significantly increased blood leukocytes and neutrophils counts, the peroxidase score and the levels of Gr1^+^, Gr1^high^ and Gr1^low^ cells (Figure S2, Figure 2). BCD+LrN group reached similar leukocytes, neutrophils and Gr1^+^ cells numbers than those observed in the WNC mice, while these parameters in the BCD+LrO group were higher than in the well-nourished controls (Figure 2). In addition, blood Gr1^low^ cells and peroxidase activity in both BCD+LrO and BCD+LrN mice were significantly higher than in WNC mice (Figure 2). After the challenge with pneumococci, blood CD34^+^ cells were increased in all the experimental groups in relation to basal levels. No difference was observed between CD34^+^ blood cells in BCD and BCD+LrN mice. Notably BCD+LrO mice showed significantly higher numbers of blood CD34^+^ cells when compared with the other experimental groups (Figure 2).

BCD mice showed higher levels of BAL IL-1β, IL-6, IFN-γ and serum TNF-α and IL-6 than MNC mice; however these cytokines did not reach the values of the WNC group (Figure 3). Cytokine responses to the pneumococcal infection in BCD+LrO and BCD+LrN mice were similar to WNC mice. No change was observed in levels of BAL TNF-α, IL-1β, IL-6 and serum IL-1β, IL-6 between those groups (Figure 3). On the contrary, levels of serum IL-6 and BAL and serum IFN-γ in BCD+LrO and BCD+LrN mice were significantly higher when compared with the WNC group. In addition, levels of BAL and serum IL-10 were normalized with BCD+LrO and BCD+LrN treatments, an effect that was not achieved with the BCD treatment (Figure 3).

### Protein-malnutrition significantly impairs emergency granulopoiesis during pneumococcal infection

**Figure 4:**
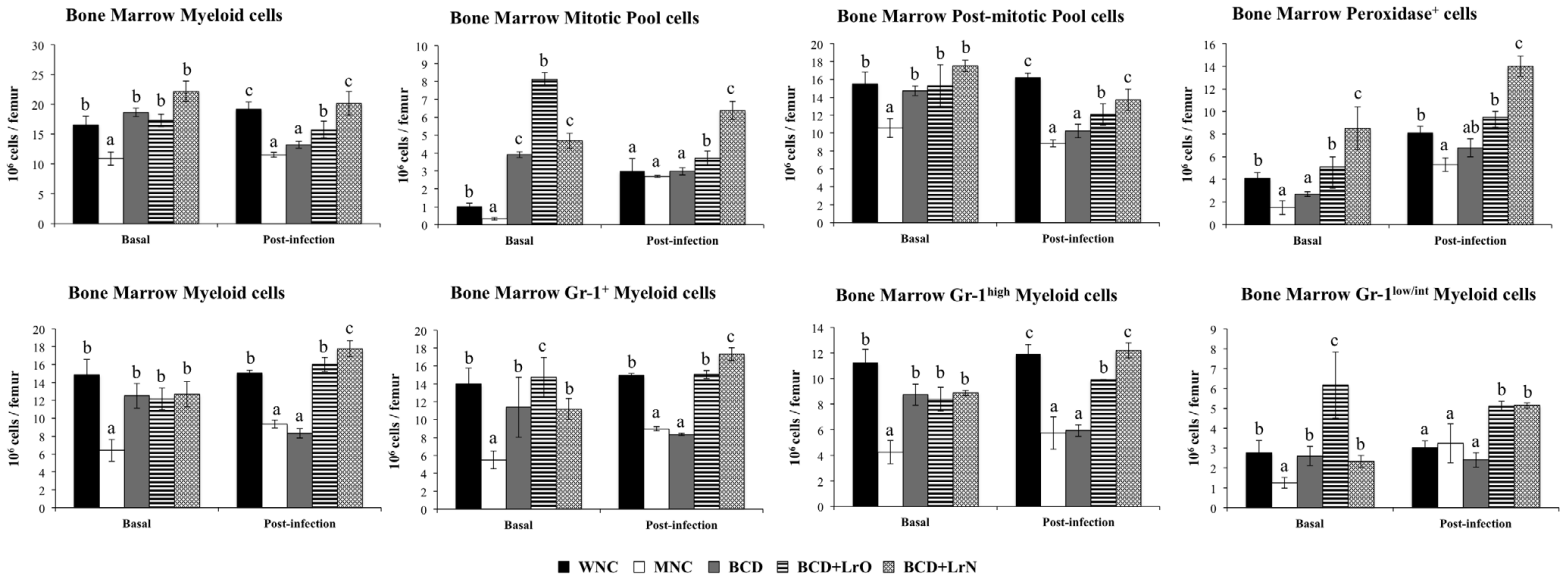
Myeloid cell populations in bone marrow. Malnourished mice were replete for 7 days with a balanced conventional diet (BD) or BD supplemented with orally or nasally administered *Lactobacillus rhamnosus* CRL1505 (BD+LrO and BD+LrN respectively) and then challenged with 10^5^ cells of *Streptococcus pneumoniae*. Malnourished (MNC) and well-nourished (WNC) mice were used as controls. Bone marrow myeloid cells, mitotic pool, post-mitotic pool, Gr1^+^ cells, Gr1^high^ cells and Gr1^low/int^ cells numbers and the score of bone marrow cells myeloperoxidase were determined before (basal) and after pneumococcal infection (day 2). Values are means for six mice per group with standard deviations represented by verticals bars. Different letters indicate significant differences at the same time point (*p*<0.05) considering a<b<c.

Resistance against *S. pneumoniae* infection was significantly reduced in MNC mice since the levels of lung and blood bacterial cell counts were higher than those detected in WNC mice (Figure S3). Changes in emergency granulopoiesis were next studied. A significant reduction of bone marrow cells (Figure S2) together with a reduction of myeloid cells (Figure 4) was observed in MNC mice by using conventional hematological technics. In addition, we detected a significant reduction of all the myeloid cells populations: mitotic pool cells (myeloblast, promyielocytes, myelocytes) and post-mitotic pool cells (metamyelocytes, band cells, neutrophils) in MNC mice (Figure 4). These data were confirmed by flow cytometry, which showed a significant reduction in the numbers of bone marrow Gr-1^+^, Gr-1^high^ and Gr-1^low/int^ cells (Figure 4). Challenge with *S. pneumoniae* induced a small increase of myeloid cells with increases in mitotic pool cells and without changes in the number of post-mitotic cells in WNC mice (Figure 4). In addition, no changes were detected in the numbers of bone marrow Gr-1^+^, Gr-1^high^ and Gr-1^low/int^ cells after the infection (Figure 4). In MNC mice however, this response was significantly modified since all the parameters studied in bone marrow were significantly lower than WNC mice, with the exception of mitotic pool cells and Gr-1^low/int^ cells which were not different from the WNC group (Figure 4). These results correlated with the peroxidase activity in bone marrow, which is a cytochemical marker of myeloid cells. The numbers of peroxidase positive cells in MNC mice were lower than in WNC animals before and after the respiratory infection (Figure 4).

**Figure 5:**
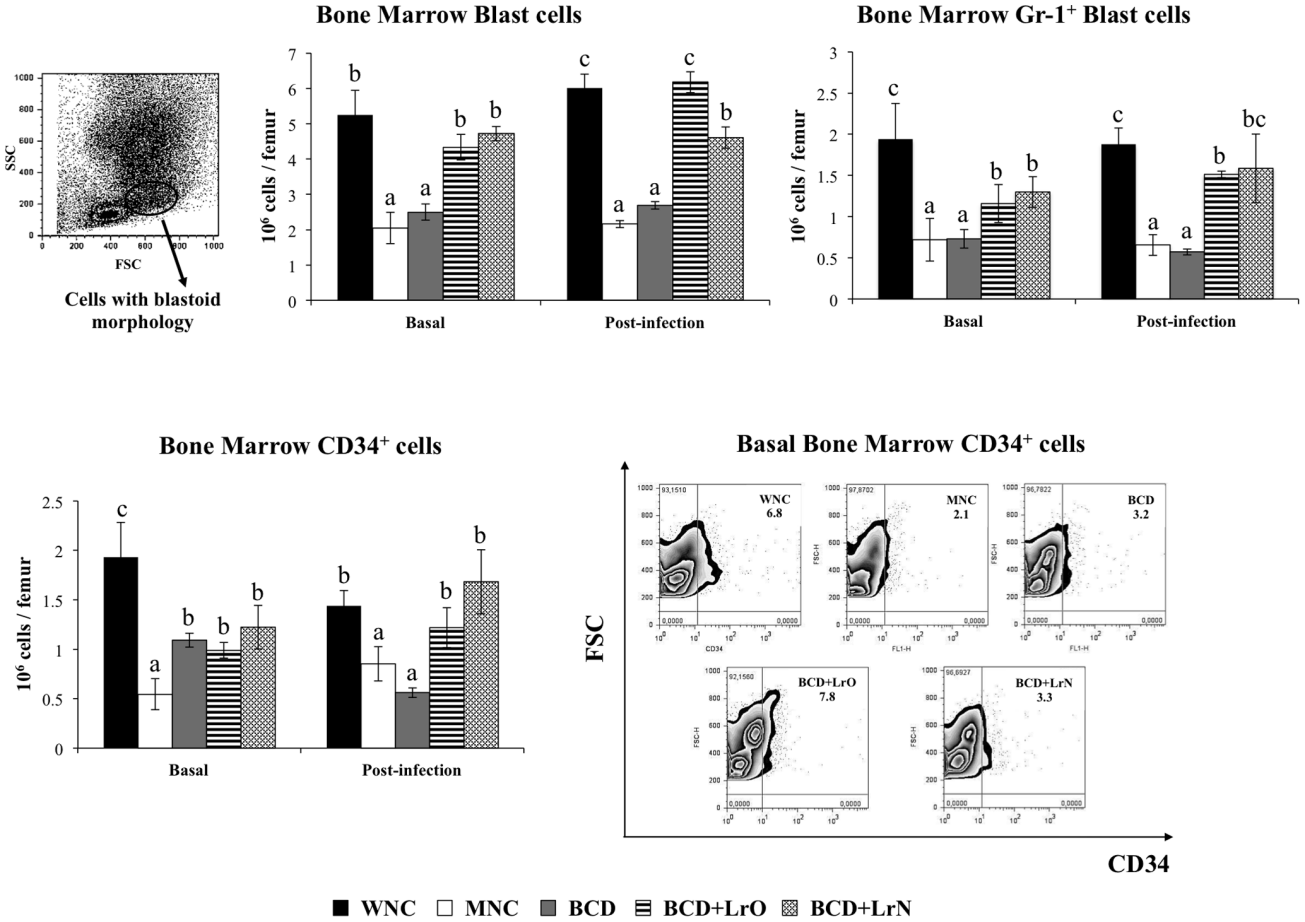
Blast cells in bone marrow. Malnourished mice were replete for 7 days with a balanced conventional diet (BD) or BD supplemented with orally or nasally administered *Lactobacillus rhamnosus* CRL1505 (BD+LrO and BD+LrN respectively) and then challenged with 10^5^ cells of *Streptococcus pneumoniae*. Malnourished (MNC) and well-nourished (WNC) mice were used as controls. Bone marrow blast cells and the expression of CD34 and Gr1 markers in those cells were determined before (basal) and after pneumococcal infection (day 2). Values are means for six mice per group with standard deviations represented by verticals bars. Different letters indicate significant differences at the same time point (*p*<0.05) considering a<b<c.

The numbers of bone marrow cells with blastoid morphology were then examined as well as the expression of Gr-1 in that population. Malnutrition significantly reduced both parameters (Figure 5). In addition, CD34 was studied as a progenitor cell marker [16]. Bone marrow CD34^+^ cells in MNC mice were also diminished in comparison with the WNC group (Figure 5). Pneumococcal infection did not induce changes in these cell populations since the levels of bone marrow cells with blastoid morphology, their expression of Gr-1 and CD34^+^ cells in both WNC and MNC mice were equal to basal levels (Figure 5).

**Figure 6:**
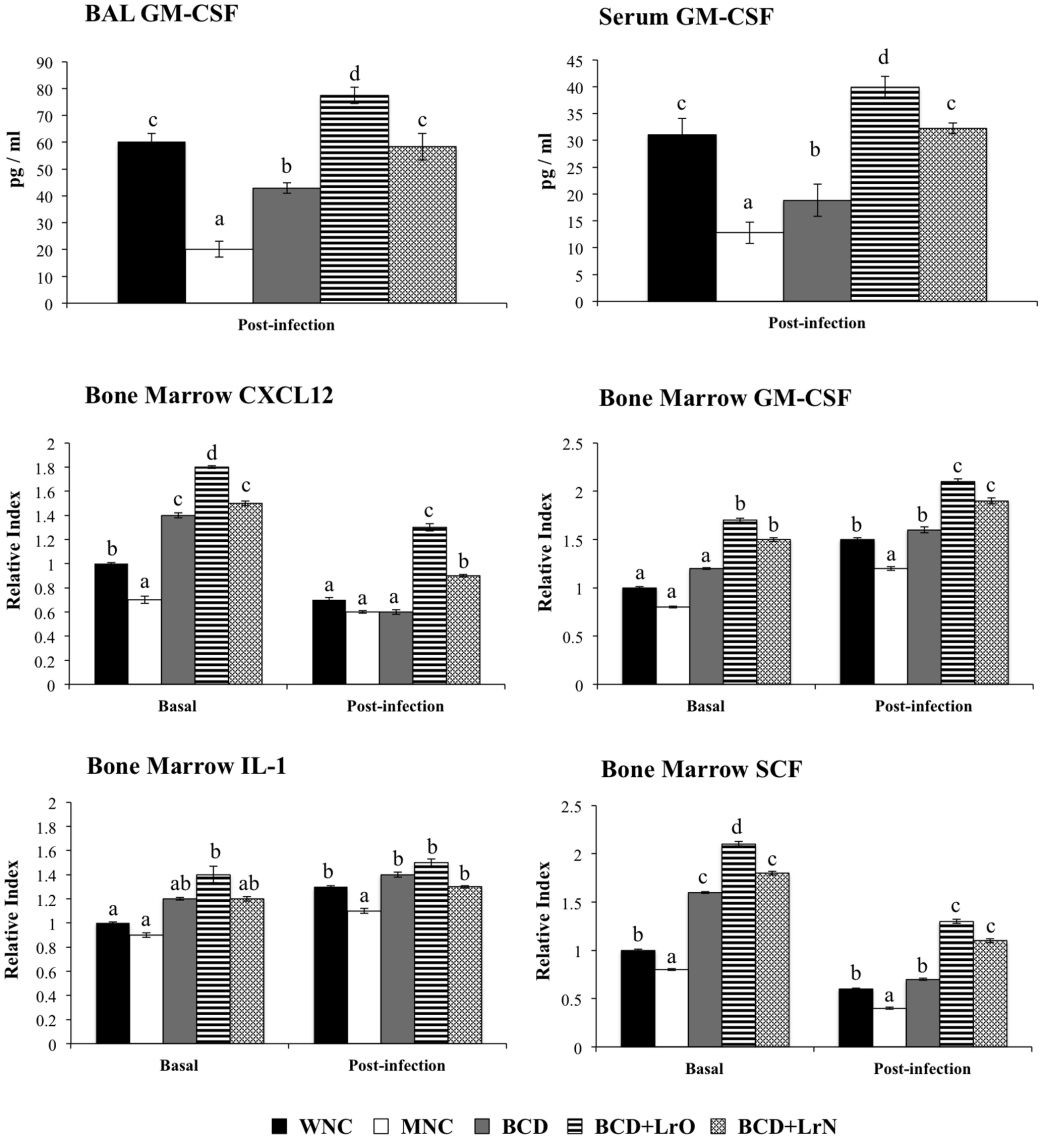
Cytokines and hematopoietic grow factors expression in bone marrow. Malnourished mice were replete for 7 days with a balanced conventional diet (BD) or BD supplemented with orally or nasally administered *Lactobacillus rhamnosus* CRL1505 (BD+LrO and BD+LrN respectively) and then challenged with 10^5^ cells of *Streptococcus pneumoniae*. Malnourished (MNC) and well-nourished (WNC) mice were used as controls. Levels of mRNA of bone marrow GM-CSM, GM-CSF, CXCL12, GM-CSF, IL-1 and SCF were determined before (basal) and after pneumococcal infection (hours 12 and 48). Values are means for six mice per group with standard deviations represented by verticals bars. Different letters indicate significant differences at the same time point (*p*<0.05) considering a<b<c.

The production of hematopoietic grow factors in infected lungs and blood as well as their expression in bone marrow was also investigated. Concentrations of GM-CSF were determined in serum and BAL of WNC and MNC mice. GM-CSF in serum and BAL of uninfected animals was not detected by a commercially available ELISA kit (data not shown). However, levels of GM-CSF were significantly increased in serum and BAL of WNC and MNC mice after the challenge with *S. pneumoniae* (Figure 6). The levels of this hematopoietic grow factor were significantly lower in MNC mice when compared with the WNC group. In addition, the expression of CXCL12, GM-CSF, IL-1 and SCF in bone marrow was studied. Protein-malnutrition significantly reduced mRNA levels of CXCL-12 and SCF in bone marrow while GM-CSF and IL-1 expression was not affected (Figure 6). Challenge with the respiratory pathogen significantly reduced the expression of CXCL12 and SCF and increased the expression of GM-CSF and IL-1 in bone marrow of WNC mice (Figure 6). Expression of SCF, GM-CSF and IL-1 in bone marrow of MNC mice was also reduced by the pneumococcal infection; however, mRNA levels of these hematopoietic grow factors were significantly lower than those observed in WNC (Figure 6). Differently, CXCL12 expression was not modified by the infectious process in MNC mice (Figure 6).

### *L. rhamnosus* CRL1505 administration improves emergency granulopoiesis during pneumococcal infection

Finally, the effect of dietary treatments on the resistance against the pneumococcal infection and emergency granulopoiesis was studied. Resistance against *S. pneumoniae* infection in BCD mice was not different from the MNC group. However, BCD+LrO and BCD+LrN mice showed an improved resistance against the pneumococcal infection when compared to the BCD (Figure S3). Administration of BCD to malnourished mice induced the normalization of total bone marrow cells (Figure S2), myeloid and post-mitotic pool cells counts and the number of bone marrow Gr-1^+^, Gr-1^high^ and Gr-1^low/int^ cells (Figure 4). However, bone marrow peroxidase positive cells in BCD mice were not different from that observed in MNC, while mitotic pool cells were higher than WNC mice (Figure 4). Both BCD+LrO and BCD+LrN treatments normalized the levels of total cells count (Figure S2), myeloid and post-mitotic pool cells and the number of Gr-1^+^ and Gr-1^high^ cells in the bone marrow (Figure 4). Furthermore, BCD+LrO treatment normalized peroxidase cells count and induced a significant increase of mitotic pool cells counts and the number of Gr-1^low/int^ cells when compared with the WNC group (Figure 4). On the contrary, BCD+LrN treatment normalized bone marrow mitotic pool cells counts and the number of Gr-1^low/int^ cells while induced a significant increase of peroxidase positive cells when compared with WNC mice (Figure 4).

After the challenge with *S. pneumoniae* all the parameters studied in bone marrow of BCD mice were not different from those observed in the MNC group, with the exception of peroxidase positive cells that reached similar values to WNC mice (Figure 4). BCD+LrO mice showed total bone marrow cell count, myeloid cells and peroxidase values similar to those detected in WNC mice (Figure 4). In addition, mitotic pool cells and Gr-1^low/int^ cells in BCD+LrO mice were higher than WNC mice, while post-mitotic pool cells and Gr-1^high^ cells reached values between those observed in MNC and WNC groups (Figure 4). BCD+LrN mice showed values of total bone marrow cells, myeloid, post mitotic pool and Gr-1^high^ cells numbers similar to those detected in WNC mice (Figure S2, Figure 4). However, mitotic pool and Gr-1^low/int^ cells numbers and peroxidase positive cells counts in BCD+LrN were significantly higher than the WNC group (Figure 4).

BCD treatment was not able to improve bone marrow cells with blastoid morphology or the expression of Gr-1 in that population since the number of these cells was not different from the MNC mice (Figure 5). However, BCD mice showed improved numbers of CD34^+^ cells although the treatment was not able to normalize this population of cells (Figure 5). Both BCD+LrO and BCD+LrN treatments normalized the numbers of bone marrow cells with blastoid morphology. In addition, both dietary treatments improved the expression of Gr-1 bone marrow cells with blastoid morphology and the numbers of CD34^+^ cells although the values of these parameters did not reach those observed in WNC mice (Figure 5).

As described for WNC and MNC mice, the pneumococcal infection did not induce changes in these cell populations since the levels of bone marrow cells with blastoid morphology, their expression of Gr-1 and CD34^+^ cells in BDC, BCD+LrO and BCD+LrN mice were equal to basal levels (Figure 5).

The levels of serum and BAL GM-CSF after the challenge with *S. pneumoniae* in BCD and BCD+LrN were not different from the MNC and the WNC mice respectively (Figure 6). In addition, serum and BAL GM-CSF levels in BCD+LrO were significantly higher than those observed in WNC mice (Figure 6). All the dietary treatments increased the expression of CXCL12, GM-CSF, IL-1 and SCF in bone marrow. In BCD mice levels of CXCL12, IL-1 and SCF were higher than in WNC mice while GM-CSF expression was not different from the well-nourished controls (Figure 6). The four hematopoietic grow factors were significantly upregulated in the bone marrow of BCD+LrO and BCD+LrN mice showing levels that were significantly higher than the WNC group (Figure 6). Moreover, the levels of the four hematopoietic grow factors were significantly higher in BCD+LrO mice when compared with the BCD+LrN group (Figure 6). Challenge with the respiratory pathogen significantly reduced the expression of CXCL12 and SCF and increased the expression of GM-CSF and IL-1 in bone marrow of BCD, BCD+LrO and BCD+LrN mice (Figure 6). Expression of the four hematopoietic grow factors in BCD was not different from the WNC group. In addition, the expression of IL-1 in both BCD+LrO and BCD+LrN mice was similar to the WNC mice (Figure 6). However, mRNA levels of CXCL12, SCF and GM-CSF were significantly higher than WNC mice in in both BCD+LrO and BCD+LrN groups.

## Discussion

In the present work we studied the effect of malnutrition on the hemato-immune response to the respiratory challenge with *S. pneumoniae* and evaluated whether the dietary recovery with an immunobiotic strain has a beneficial effect in such response. Three important conclusions can be inferred from the results presented in this work: a) protein-malnutrition significantly impairs the emergency myelopoiesis in response to pneumococcal infection; b) repletion of malnourished mice with treatments including an immunobiotic strain can accelerate the recovery of granulopoiesis and improve innate immunity and; c) the immunological mechanisms involved in the protective effect of immunobiotics vary according to the route of administration.

### a) Protein-malnutrition significantly impairs the emergency granulopoiesis triggered in response to pneumococcal infection

**Figure 7:**
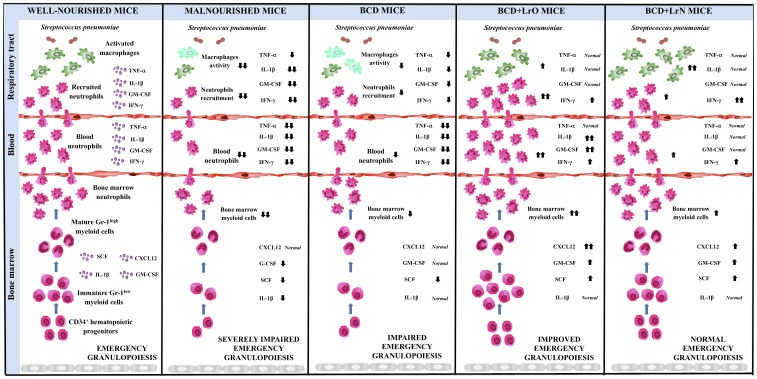
Proposed mechanisms for the effects of malnutrition and repletion treatments on emergency granulopoiesis. Malnourished mice (MNC), well-nourished mice (WNC), malnourished mice replete with a balanced conventional diet (BD) or BD supplemented with orally or nasally administered *Lactobacillus rhamnosus* CRL1505 (BD+LrO and BD+LrN respectively) and then challenged with *Streptococcus pneumoniae*. Tumor necrosis factor alpha (TNF-α); Interleukin 1 beta (IL-1β); Granulocyte-macrophage colony-stimulating factor (GM-CSF); Interferon gamma (INF-γ); colony-stimulating factor (CSF); C-X-C motif chemokine 12 (CXCL-12).

Studies on murine models of *S. pneumoniae* pneumonia using immunocompetent mice described that the respiratory pathogen increases the production of inflammatory mediators such as TNF-α, IL-1, IL-6, LTB4, NO, KC (CXCL1), MIP-2 (CXCL2), MCP-1 (CCL2) and RANTES (CCL5) and induces neutrophils and macrophages infiltration in the lung [17], [18]. These changes in pro-inflammatory mediators and cells in lungs were found in WNC mice in the present work. WNC mice upregulated the production of TNF-α, IL-1β and IL-6 after pneumococcal challenge and recruited neutrophils into the respiratory tract. Neutrophils constitute the first line of phagocytic defense and quickly migrate to infected tissue sites to eliminate pathogenic microbes. Due to the relatively short life span of neutrophils, the bone marrow continuously produces these phagocytes from hematopoietic precursors to maintain homeostatic levels in blood. During infections, the bone marrow production of neutrophils is significantly enhanced to reinforce host defense against invading pathogens. Emergency granulopoiesis, which is the hematopoietic response to infection, is characterized by mobilization of neutrophils from the bone marrow, leading to leukocytosis and neutrophilia. At the same time, granulocytic precursors accelerate cell cycle progression to replenish mature neutrophils in bone marrow [14]–[19]. In this regard, challenge with *S. pneumoniae* significantly increased the numbers of Gr-1^high^ mature and Gr-1^low^ immature myeloid cells in bone marrow of WNC mice that correlated with the observed neutrophilia. On the contrary, MNC mice showed significantly lower levels of BAL and serum TNF-α, IL-1β and IL-6 and reduced number of neutrophils in BAL after the challenge with pneumococci. Moreover, MNC mice were unable to expand Gr-1^high^ mature and Gr-1^low^ immature myeloid cells in bone marrow in response to the respiratory pathogen which resulted in a reduced number of neutrophils in blood (Figure 7). Therefore we have demonstrated in this work that protein-malnutrition significantly reduces the capacity to recruit neutrophils into the infected lungs and that this effect is related to the impairment in granulopoiesis.

Several factors could be involved in the impairment of emergency granulopoiesis in malnourished mice. Malnutrition affects tissues that have a high turnover rate and cell proliferation as the hematopoietic tissue and induces an impairment of blood cells production, leading to hypoplasia and structural changes of bone marrow [8], [15], [20]. Previously, we observed that malnutrition induces severe histological alterations in bone marrow, showing a decrease of hematopoietic space with an increase of extracellular matrix [15]. These histological changes could be responsible of the impairment of the hematopoietic microenvironment, which could affect the interactions between cells and cellular signaling. On the other hand, the flow cytometer analysis showed a reduction of bone marrow CD34^+^ cells in malnourished mice. This cell marker is expressed in Short Term-Haematopoietic Stem Cells, (ST-HSC) that have the potential to produce all blood cell types and with a limited capacity for self-renewal [21] and in Common Myeloid Progenitors (CMP) capable to produce myeloid cells (granulocytes, monocytes, dendritic cells) [21]. Then, it would be possible that the reduction of CD34^+^ cells, found in malnourished mice was related with the decrease of myeloid cells and the hipocellularity of the bone marrow.

The emergency granulopoiesis requires the coordinated expression of many different cytokines and chemokines as well as local hematopoietic growth factors in the bone marrow. The chemokine CXCL12 (stromal derived factor-1, SDF-1), through interaction with its major receptor CXCR4, plays a key role in controlling neutrophil homeostasis in the steady-state [22], [23]. Recent evidence also suggests that CXCR4 may play an important role in emergency granulopoiesis. Treatment with G-CSF results in a decrease in CXCL12 expression in the bone marrow [24]. These observations suggest the hypothesis that disruption of CXCR4 signaling is a key step mediating neutrophil release by G-CSF. In the present work we demonstrated that the respiratory challenge with *S. pneumonie* increased levels of G-CSF mRNA and reduced CXCL12 expression in the bone marrow of WNC mice which is in line with the results of Delano et al., [25], that showed, using a model of polymicrobial sepsis, that bone marrow CXCL12 mRNA abundance and specific CXCL12 levels were reduced during infections. Moreover, the authors demonstrated that changes in the pattern of CXCL12 signaling during infections are essential for mobilization of neutrophils from the bone marrow and for host survival. We observed that MNC mice did not modify bone marrow CXCL12 expression in response to pneumococcal infection. Then, considering that it is known that the CXCL12–CXCR4 axis is essential for hematopoietic stem cells homing into the bone marrow [26], the lack of changes in CXCL12 expression in MNC mice could be a mechanism triggered to preserve the stem cell progenitors in the bone marrow of this group.

It is well established that GM-CSF controls homeostatic and emergency development of granulocytes. In addition, IL-1 has a major role in the regulation of bone marrow stromal cell function to support hematopoiesis [27]. Then both GM-CSF and IL-1 play necessary roles in the support of neutrophilia induced by infection or inflammation by expanding both pluripotent and myeloid progenitor compartments to accelerate granulopoiesis [28]. Therefore, the impaired expression of both GM-CSF and IL-1 in bone marrow would also contribute to the altered emergency granulopoietic response in MNC mice.

In addition, emergency granulopoiesis is regulated by multiple hematopoietic growth factors that are produced in the infected tissue. It was observed a small and transient peak of GM-CSF in lung of immunocompetent mice after pneumococcal infection which occurred during the early phase of neutrophil infiltration [29]. Although several cytokines can modulate neutrophil production, G-CSF and GM-CSF are the primary regulators of steady-state and emergency granulopoiesis. In addition to regulating neutrophil output, signaling through G-CSF and GM-CSF also controls neutrophil survival, function and egress from bone marrow. Consequently, mice lacking G-CSF or GM-CSF are severely neutropenic [19], [30]. In line with these findings we observed in our experimental model that challenge with pneumococci significantly increase BAL and serum GM-CSF levels of WNC mice while significantly lower levels were detected in MNC mice. Therefore, the reduced capacity of MNC mice to produce appropriate amounts of GM-CSF could also explain the reduced response of bone marrow to the infectious challenge.

### b) Dietary recovery of malnourished mice with *L. rhamnosus* CRL1505 improves granulopoiesis and innate immunity

In previous works we demonstrated that the recovery of the respiratory immunity in malnourished mice was reduced from 21 days to 7 days when the BCD administration was supplemented with some immunobiotic strains such as *L. casei* CRL431 or *L. rhamnosus* CRL1505 [5]–[9]. In the present work we showed that the administration of the BCD during 7 days is not enough to induce the normalization of the emergency granulopoiesis in response to pneumococcal infection and that the supplementation of the BCD with *L. rhamnosus* CRL1505 provided significant advantages in the recovery of hematopoiesis (Figure 7). According to our results, the nasal treatment with *L. rhamnosus* CRL1505 was as efficient as oral treatment [15] to recover the architecture of bone marrow tissue altered by the malnutrition (Figure S4). In this regard, we observed a significant recovery of the bone marrow cellularity and sub-endosteal epithelium. Considering that hematopoiesis is the result of the interactions between progenitors stem cells and the microenvironment that comprised the osteoblastic and vascular niches [31]–[33], it is possible that the recovery observed in bone marrow is important in the reestablishment of the hematopoiesis in BCD+LrO y BCD+LrN groups. In addition, *L. rhamnosus* CRL1505 administration was able to induce an increase in bone marrow proliferating cells (mitotic pool cells), Gr-1^high^ mature myeloid cells and neutrophils. Although the underlying mechanisms were not elucidated, there is evidence that, during colonization of gut mucosa by commensal bacteria or probiotics, peptidoglycan is constantly turned over and either excreted or translocated across the intestinal mucosa into the circulation. Furthermore, peptidoglycan can accumulate in the bone marrow since it was reported that this molecule could be detected in the neutrophil fraction [34]. Then, these data showed a mechanism for systemic immunomodulation by the microbiota and provide a direct example of a probable mechanism for a probiotic activity of the microbiota, demonstrating that translocated microbial products benefit the host by enhancing systemic innate immune function.

On the other hand, it was described that some LAB can influence interleukins levels in blood [35], which agree with our results demonstrating the capacity of LAB to normalize levels of TNF-α, IL-1β, IL-4, IL-6 and IL-10 in malnourished mice [4]. Taking into account that new evidence indicates that hematopoietic progenitors respond to cytokine signaling directly and that IFN-α/β, IFN-γ, and TNF-α directly regulate hematopoietic progenitors function [36], it is probably that the changes in interleukins levels induced by LAB could influence on the normalization of hematopoiesis. Moreover, in this work we demonstrated for the first time that dietary supplementation with probiotics can modulate the production of the hematopoietic growth factor GM-CSF in infected lungs and its expression in the bone marrow. In response to infectious stimuli, bone marrow B lymphocytes and mature granulocytes are mobilized to the peripheral circulation through a CXCR4/CXCL12-associated mechanism. We have demonstrated before that the treatment with *L. rhamnosus* CRL1505 accelerates the recovery of B lymphopoiesis [15]. Here, we found a recovery of the mielopoyesis in BCD+LrO and BCD+LrN groups. Then, the signaling axis CXCR4/CXCL12 would be important in the recovery of hematopoiesis induced by *L. rhamnosus* CRL1505. Further studies of the influence of immunobiotics in the CXCR4/CXCL12 signaling in bone marrow are an interesting topic for future investigations.

### c) The immunological mechanisms involved in the protective effect of immunobiotics would vary according to the route of administration

The results presented here are in line with our previous publications in demonstrating that nasal priming with immunobiotics is more efficient than oral administration to improve resistance against respiratory pathogens, since lower pnuemococcal cell counts were found in lungs and blood of BCD+LrN mice when compared to the BCD+LrO group. In addition, comparative studies using BCD+LrO and BCD+LrN showed that both treatments were efficient for improving the recovery of bone marrow myelopoiesis (Figure 7). However, oral administration of *L. rhamnosus* CRL1505 was more effective than nasal priming to improve emergency granulopoiesis in malnourished mice. Then, additional mechanism(s) should explain the higher efficiency of the nasal priming to protect against pneumococcal infection.

It was described that different immunization routes (intranasal and oral) can induce generalized immune responses, although the relative representation of dominant antibody isotypes may vary. Nevertheless, nasal immunization appears to induce secretory IgA immunity in a broader range of mucosal tissues than oral vaccination [37]–[40]. In this context, it is probable that the oral administration of *L. rhamnosus* CRL1505 could stimulate local and systemic immunity, while the nasal priming could induce systemic immunity and stimulate especially the respiratory mucosal tissue, which could be an advantage in the protection against respiratory pathogens. Therefore, the comparative study between oral and nasal treatments addressed in this work, gives important information for the selection of the appropriate route for immunobiotics administration. Moreover, the results obtained here are critical for the future application of LAB as mucosal adjuvants, which could permit the use of low immunogenic antigens when administered by mucosal routes.

## Conclusions

There are some reviews about the effect of immunobiotics on innate immunity in immunocompetent and immunocompromised hosts, and about the mechanisms involved in their action at intestinal and respiratory levels [4], [12], [41], [42]. However, there are very few reports about the influence of the probiotic administration on hematopoiesis. Our laboratory has made some important findings in this sense and although there is still a long way to go in the study of the interactions between immunobiotic and hematopoiesis, the results obtained so far, demonstrated that is possible to reverse the alterations of myeloid and lymphoid progenitors in malnourished mice by using a dietary supplementation with an appropriate immunobiotic LAB [8], [11], [15]. In the present study we demonstrated that dietary recovery of malnourished mice with oral or nasal administration of *L. rhamnosus* CRL1505 improves emergency granulopoiesis and that CXCR4/CXCR12 signaling would be involved in this effect. Then, the results summarized here are a starting point for future research and open up broad prospects for future applications of immunobiotics.

## Materials and Methods

### Lactic acid bacterium

*Lactobacillus rhamnosus* CRL1505 was obtained from the CERELA culture collection (Chacabuco 145, San Miguel de Tucumán, Argentina). The culture was kept freeze-dried and then rehydrated using the following medium: peptone 15.0 g; tryptone 10.0 g; meat extract 5.0 g; distilled water 1l, pH 7. It was cultured for 8 h at 37°C (final log phase) in Man-Rogosa-Sharpe broth (MRS, Oxoid). The bacteria were harvested through centrifugation at 3000 *g* for 10 min and washed three times with sterile 0.01 mol/l phosphate buffer saline (PBS), pH 7.2.

### Animals and feeding procedures

The experimental protocols were approved by the Ethical Committee of Animal Care from the Reference Centre for Lactobacilli (CERELA-CONICET, Tucuman, Argentina), under the protocol number LI-2013-02.

Male 3-week-old Swiss albino mice were obtained from the closed colony kept at CERELA. They were housed in plastic cages at 25°C. The assays were performed in six mice per group for each time point (in three independent experiments). Weaned mice were fed with a protein-free diet (PFD) for 21 days, and the animals that weighed 45–50% less than well-nourished mice were selected for the experiments [5].

Malnourished mice were separated in three groups for repletion treatment: i) balanced conventional diet (BCD) for 7 consecutive days (BCD group); ii) BCD for 7 days with oral *L. rhamnosus* CRL1505 supplementation (10^8^ CFU/mouse/day) during last 5 days of the treatment (BCD+LrO group); iii) BCD for 7 days with nasal *L. rhamnosus* CRL1505 supplementation (10^8^ CFU/mouse/day) during last 2 days of the treatment (BCD+LrN group). The administration of 10^8^ cells of *L. rhamnosus* CRL1505 was previously selected as the optimal dose able to improve protection against *S. pneumoniae* and *S. typhimurium* in immunocompetent mice [9]. The malnourished control (MNC) group received only PFD and well-nourished control (WNC) mice consumed BD *ad-libitum*.

### Pneumococcal infection

The experimental animal model of pneumococcal respiratory infection was used as previously described [5], [6]. *Streptococcus pneumoniae* serotype 14 (ANLIS, Argentina) was obtained from the respiratory tract of a patient from the Children's Hospital, Tucuman, Argentina [5], [6]. Briefly, the different experimental groups of mice were nasally challenged with *S. pneumoniae* (10^5^ CFU/ml in PBS) at the end of each treatment (day 8th). WNC and MNC (without repletion treatment) groups were infected equally. Animals were sacrificed at day 0 (before challenge) and at different days after infection.

### Bacterial cell counts in lung homogenates and blood

Mice were sacrificed on days 2 after challenge with *S. pneumoniae* and their lungs were excised, weighed and homogenized in 5 ml of sterile 0.1% peptone water [5], [6]. Homogenates were diluted appropriately, plated in duplicate on blood agar and incubated 18 h at 37°C. The results were expressed as log of CFU/g lung. Progression of bacterial growth to the bloodstream was monitored by sampling blood obtained through cardiac puncture and plating on blood agar. Results were reported as CFU/ml.

### Cytokine concentrations in bronchoalveolar lavage (BAL) and serum

Tumour necrosis factor (TNF)-α, interleukin (IL)-1β, IL-6, GM-CSF, IL-10 and interferon (IFN)-γ concentrations in serum and BAL samples were measured with commercially available enzyme-linked immunosorbent assay kits following the manufacturer's recommendations (R&D Systems, MN, USA).

### Total and differential number of leukocytes in blood and BAL

Blood and BAL samples were obtained as described above on 0 and 2 days after infection. The total number of leukocytes and differential cell counts were performed as described previously [5]–[43].

### Flow cytometry in BAL

BAL cells were preincubated with antimouse CD32/CD16 monoclonal antibody (Fc block) and stained with the following antibodies from BD PharMingen: APC-Cy7 anti-mouse CD45; phycoerythrin anti-mouse Gr-1. In addition, alveolar macrophages population was detected by their positive autofluorescence in FL1 as described by [44]. In all cases, cells were then acquired on a BD FACSCaliburTM flow cytometer (BD Biosciences) and data were analyzed with FlowJo software (TreeStar). The number of cells in each population was determined by multiplying the percentages of subsets within a series of marker negative or positive gates by the total cell number determined for each tissue.

### Phagocytic cell activation

#### Nitroblue tetrazolium (NBT) test

The phagocytic bactericidal activity (oxidative burst) of macrophages and neutrophils was measured using the NBT reduction test (catalogue no. 840-W, Sigma-Aldrich Co.) in the pellet of BAL. NBT was added to each sample with (positive control) or without addition of the bacterial extract; then samples were incubated at 37°C for 20 min. In presence of oxidative metabolites, NBT (yellow) is reduced to formazan, which forms a blue precipitate. Smears were prepared; after staining, samples were examined under a light microscope for blue precipitates. A hundred cells were counted and NBT positive (+) cells were determined.

#### Washburn test

The measurement of myeloperoxidase activity of blood neutrophils was performed as described previously [5]. Blood cells were graded as negative or weakly, moderately, or strongly positive and were used to calculate the score.

### Cells in the bone marrow

The studies in bone marrow were performed at the end of repletion period (day 0) and day 2 after challenge. The total and differential cell counts of bone marrow samples were obtained as described previously [8]. The results were expressed as 10^6^ cells/femur. The measurement of myeloperoxidase activity of bone marrow myeloid cells was performed as described above and we reported as the number of peroxidase positive cells (10^6^ cells/femur).

### Flow cytometry in blood and bone marrow

Blood and bone marrow cells were preincubated with antimouse CD32/CD16 monoclonal antibody (Fc Block) and the expression of Gr-1 and CD34 was examined by flow cytometry. Fluorescein isothiocyanate (FITC) rat anti-mouse CD34 antibody (RAM34 monoclonal antibody; BD Pharmingen) and phycoerythrin (PE) rat anti-mouse Gr-1 antibody (RB6-8C5 monoclonal antibody; BD Pharmingen) were used. We also studied the Light scatter characteristics of bone marrow cells. Bone marrow was gated into myeloid population gate and the area of blastoid morphology cells [45]. Data were acquired on a BD FACScalibur cytometer and analyzed using Flow Jo (Tree Star) software as described above.

### Quantitative expression analysis by real-time PCR

We performed two-step real-time quantitative PCR to characterize the expression of CXCL12, GM-CSF, IL-1β and SCF mRNAs in bone marrow. Total RNA was isolated from each sample using TRIzol reagent (Invitrogen). All cDNAs were synthesized using a Quantitect reverse transcription (RT) kit (Qiagen, Tokyo, Japan) according to the manufacturer's recommendations. Real-time quantitative PCR was carried out using a 7300 real-time PCR system (Applied Biosystems, Warrington, United Kingdom) and the Platinum SYBR green qPCR SuperMix uracil-DNA glycosylase (UDG) with 6-carboxyl-X-rhodamine (ROX) (Invitrogen). The following primers were used: **CXCL12** (sense: 5′-GTC CTC TTG CTG TCC AGC TC-3′; antisense: 5′-TAA TTT CGG GTC AAT GCA CA-3′); **GM-CSF** (sense: 5′-CAT CAA AGA AGC CCT GAA CC-3′; antisense: 5′-TGC ATT CAA AGG GGA TAT CAG-3′); **IL-1β** sense: 5′-GAC CTT CCA GGA TGA GGA CA-3′; antisense: 5′-AGG CCA CAG GTA TTT TGT CG-3′); **SCF** (sense: 5′-CGG GAA TCC TGT GAC TGA TAA-3′; antisense: 5′-GGC CTC TTC GGA GAT TCT TT-3′). The PCR cycling conditions were 2 min at 50°C, followed by 2 min at 95°C, and then 40 cycles of 15 s at 95°C, 30 s at 60°C, and 30 s at 72°C. The reaction mixtures contained 5 ul of sample cDNA and 15 ul of master mix, which included the sense and antisense primers. Expression of β-actin was used to normalize cDNA levels for differences in total cDNA levels in the samples.

### Statistical analysis

Experiments were performed in triplicate and results were expressed as mean values and standard deviations. A two-way analysis of variance (ANOVA) test was used to evaluate the main effects and the interactions between treatments (InfoStat, 2006). Comparisons between mean values were carried out using one-way analysis of variance and Fisher's least-significant-difference test. For these analyses, P values of <0.05 were considered significant.

## Supporting Information

Figure S1 **Innate immune cell populations in broncho-alveolar lavages (BAL).** Malnourished mice were replete for 7 days with a balanced conventional diet (BD) or BD supplemented with orally or nasally administered *Lactobacillus rhamnosus* CRL1505 (BD+LrO and BD+LrN respectively). Malnourished (MNC) and well-nourished (WNC) mice were used as controls. Leukocytes and macrophages numbers and the percentage of nitroblue tetrazolium (NBT) positive cells were determined. Values are means for six mice per group with standard deviations represented by verticals bars. Different letters indicate significant differences at the same time point (*p*<0.05) considering a<b<c.
(TIFF)

Figure S2 **Leucocytes counts in blood and bone marrow.** Malnourished mice were replete for 7 days with a balanced conventional diet (BD) or BD supplemented with orally or nasally administered *Lactobacillus rhamnosus* CRL1505 (BD+LrO and BD+LrN respectively) and then challenged with 10^5^ cells of *Streptococcus pneumoniae*. Malnourished (MNC) and well-nourished (WNC) mice were used as controls. Blood and bone marrow total leucocytes numbers were determined before (basal) and after pneumococcal infection (day 2). Values are means for six mice per group with standard deviations represented by verticals bars. Different letters indicate significant differences at the same time point (*p*<0.05) considering a<b<c.
(TIFF)

Figure S3 **Resistance to pneumococcal infection.** Malnourished mice were replete for 7 days with a balanced conventional diet (BD) or BD supplemented with orally or nasally administered *Lactobacillus rhamnosus* CRL1505 (BD+LrO and BD+LrN respectively) and then challenged with 10^5^ cells of *Streptococcus pneumoniae*. Malnourished (MNC) and well-nourished (WNC) mice were used as controls. Bacterial cell counts in lung (log CFU/g of lung) and blood (log CFU/mL) after challenge. Values are means for six mice per group with standard deviations represented by verticals bars. Different letters indicate significant differences at the same time point (*p*<0.05) considering a<b<c<d.
(TIFF)

Figure S4 **Bone marrow histological examination.** Malnourished mice were replete for 7 days with a balanced conventional diet (BD) supplemented with orally or nasally administered *Lactobacillus rhamnosus* CRL1505 (BD+LrO and BD+LrN respectively). The femur was removed; the BM fixed in paraformaldehyde, decalcified in formic acid and sodium citrate, and stained with Hematoxylin and eosin. Light micrographs, original magnification x400.
(TIFF)
