# Supplementary figures and images for: Dietary Supplementation with Lactobacilli Improves Emergency Granulopoiesis in Protein-Malnourished Mice and Enhances Respiratory Innate Immune Response
Source: PLoS One. 2014 Apr 1;9(4):e90227. doi: 10.1371/journal.pone.0090227 (PMC3972161; doi:10.1371/journal.pone.0090227)

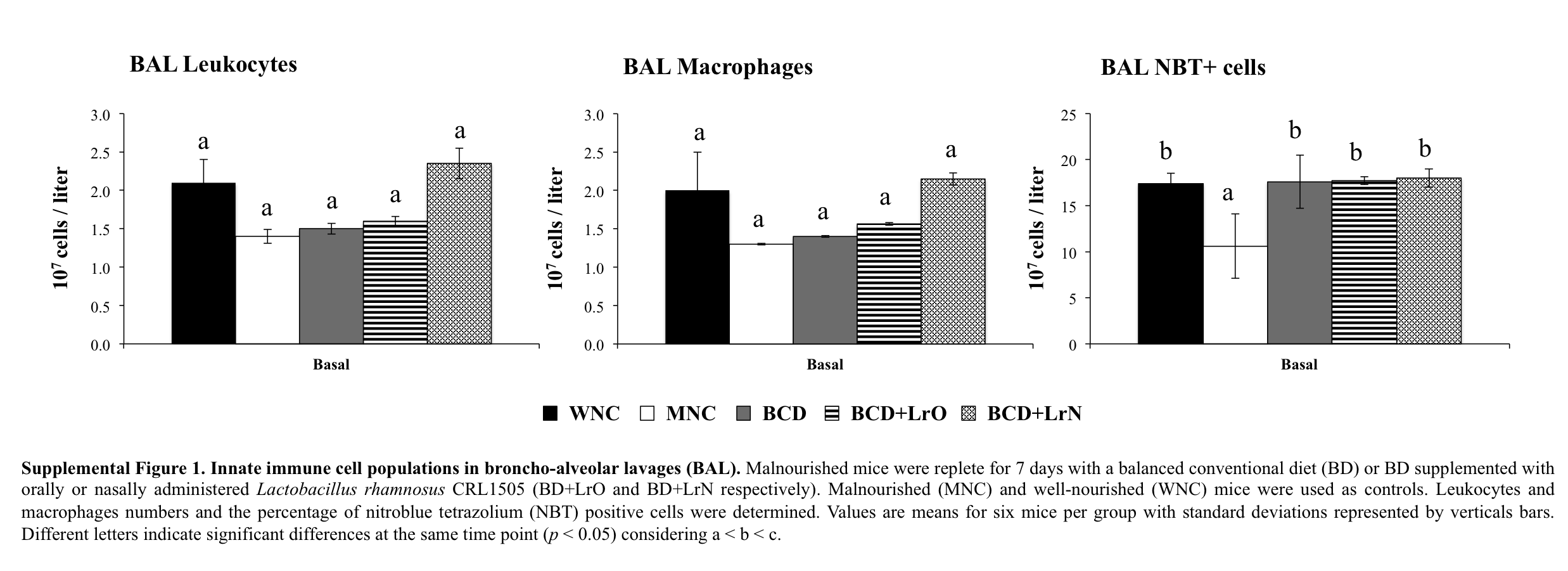

Supplement: Figure S1 — Innate immune cell populations in broncho-alveolar lavages (BAL). Malnourished mice were replete for 7 days with a balanced conventional diet (BD) or BD supplemented with orally or nasally administered Lactobacillus rhamnosus CRL1505 (BD+LrO and BD+LrN respectively). Malnourished (MNC) and well-nourished (WNC) mice were used as controls. Leukocytes and macrophages numbers and the percentage of nitroblue tetrazolium (NBT) positive cells were determined. Values are means for six mice per group with standard deviations represented by verticals bars. Different letters indicate significant differences at the same time point (p<0.05) considering a<b<c. (TIFF) [file pone.0090227.s001.tif]

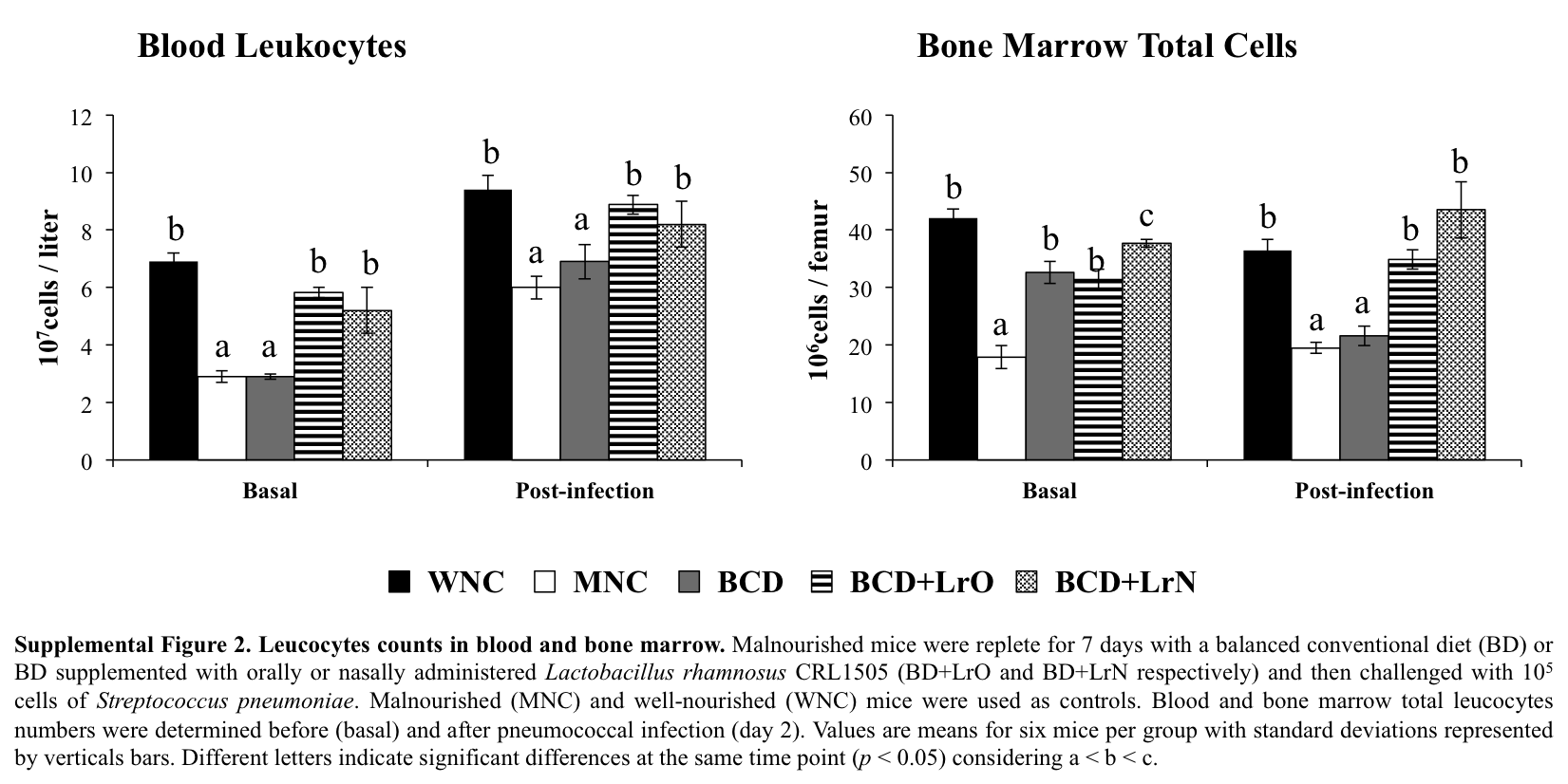

Supplement: Figure S2 — Leucocytes counts in blood and bone marrow. Malnourished mice were replete for 7 days with a balanced conventional diet (BD) or BD supplemented with orally or nasally administered Lactobacillus rhamnosus CRL1505 (BD+LrO and BD+LrN respectively) and then challenged with 105 cells of Streptococcus pneumoniae. Malnourished (MNC) and well-nourished (WNC) mice were used as controls. Blood and bone marrow total leucocytes numbers were determined before (basal) and after pneumococcal infection (day 2). Values are means for six mice per group with standard deviations represented by verticals bars. Different letters indicate significant differences at the same time point (p<0.05) considering a<b<c. (TIFF) [file pone.0090227.s002.tif]

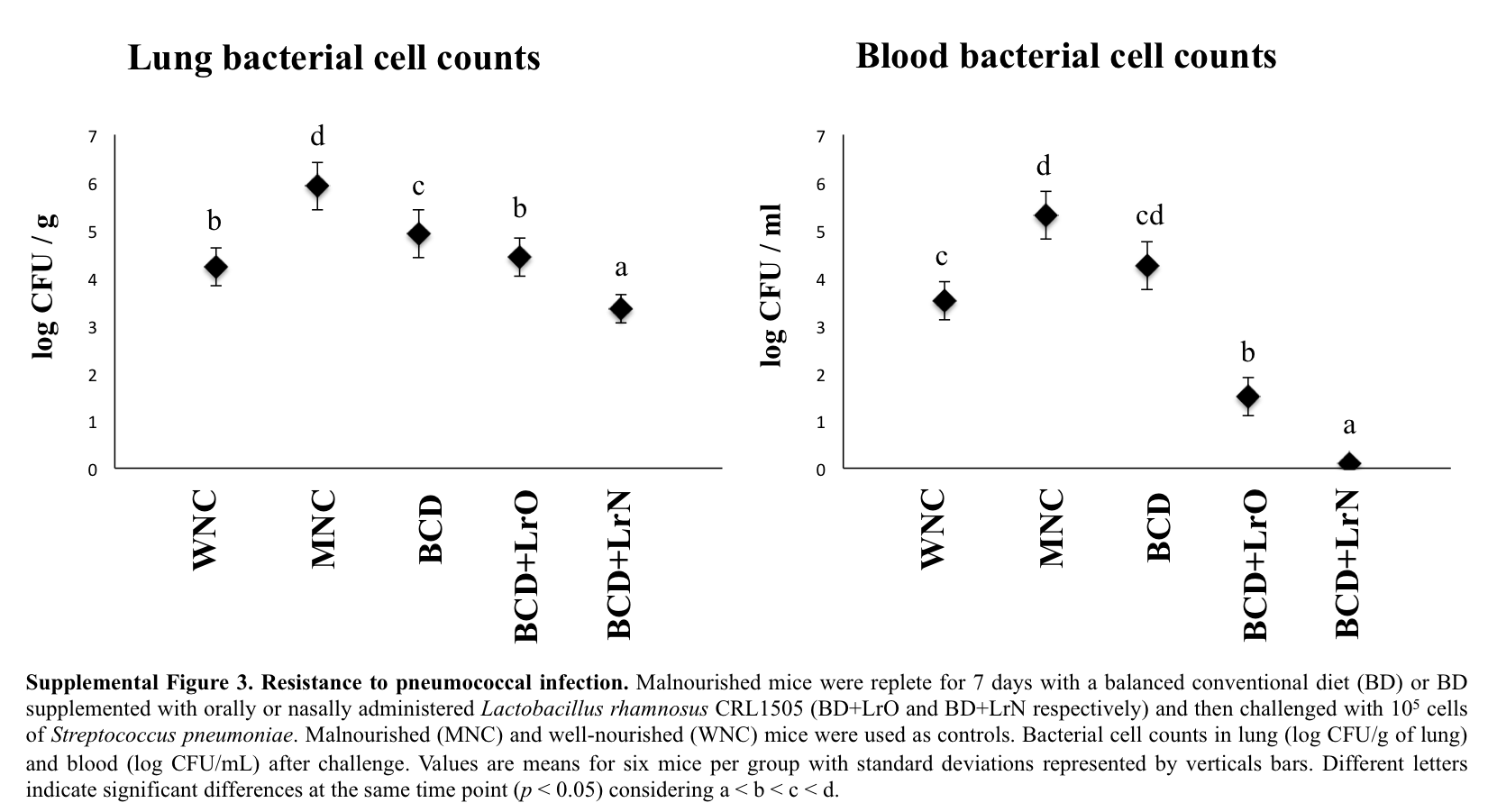

Supplement: Figure S3 — Resistance to pneumococcal infection. Malnourished mice were replete for 7 days with a balanced conventional diet (BD) or BD supplemented with orally or nasally administered Lactobacillus rhamnosus CRL1505 (BD+LrO and BD+LrN respectively) and then challenged with 105 cells of Streptococcus pneumoniae. Malnourished (MNC) and well-nourished (WNC) mice were used as controls. Bacterial cell counts in lung (log CFU/g of lung) and blood (log CFU/mL) after challenge. Values are means for six mice per group with standard deviations represented by verticals bars. Different letters indicate significant differences at the same time point (p<0.05) considering a<b<c<d. (TIFF) [file pone.0090227.s003.tif]

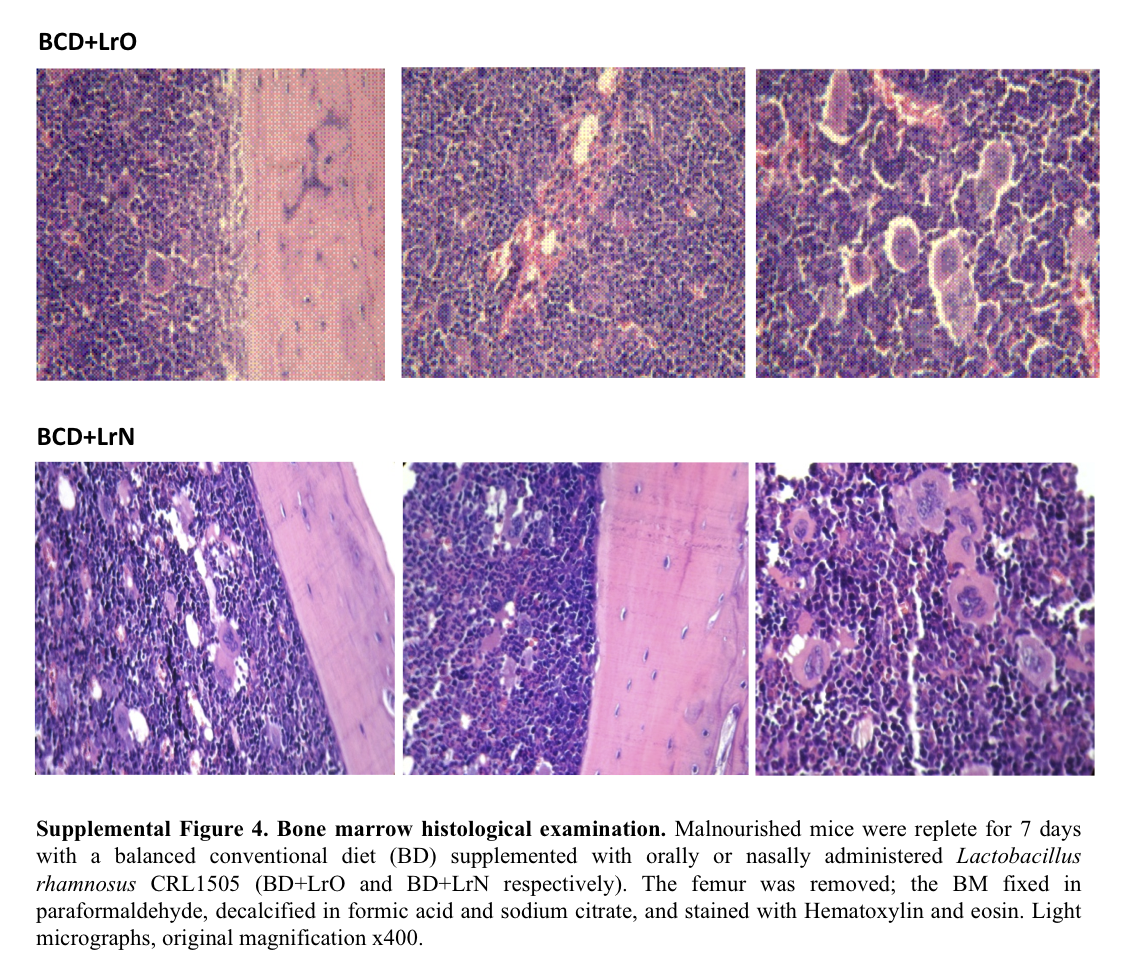

Supplement: Figure S4 — Bone marrow histological examination. Malnourished mice were replete for 7 days with a balanced conventional diet (BD) supplemented with orally or nasally administered Lactobacillus rhamnosus CRL1505 (BD+LrO and BD+LrN respectively). The femur was removed; the BM fixed in paraformaldehyde, decalcified in formic acid and sodium citrate, and stained with Hematoxylin and eosin. Light micrographs, original magnification x400. (TIFF) [file pone.0090227.s004.tif]
